# A New Era of Dental Care: Harnessing Artificial Intelligence for Better Diagnosis and Treatment

**DOI:** 10.7759/cureus.49319

**Published:** 2023-11-23

**Authors:** Aastha Mahesh Batra, Amit Reche

**Affiliations:** 1 Dentistry, Sharad Pawar Dental College and Hospital, Datta Meghe Institute of Higher Education and Research, Wardha, IND; 2 Public Health Dentistry, Sharad Pawar Dental College and Hospital, Datta Meghe Institute of Higher Education and Research, Wardha, IND

**Keywords:** collaborative approach, ethical considerations, patient engagement, treatment planning, dental diagnosis, artificial intelligence in dentistry

## Abstract

The integration of artificial intelligence (AI) into dental care holds the promise of revolutionizing the field by enhancing the accuracy of dental diagnosis and treatment. This paper explores the impact of AI in dental care, with a focus on its applications in diagnosis, treatment planning, and patient engagement. AI-driven dental imaging and radiography, computer-aided detection and diagnosis of dental conditions, and early disease detection and prevention are discussed in detail. Moreover, the paper delves into how AI assists in personalized treatment planning and provides predictive analytics for dental care. Ethical and privacy considerations, including data security, fairness, and regulatory aspects, are addressed, highlighting the need for a responsible and transparent approach to AI implementation. Finally, the paper underscores the potential for a collaborative partnership between AI and dental professionals to offer the best possible care to patients, making dental care more efficient, patient-centric, and effective. The advent of AI in dentistry presents a remarkable opportunity to improve oral health outcomes, benefiting both patients and the healthcare community.

## Introduction and background

Dental care plays a pivotal role in maintaining overall health and well-being. The oral cavity is essential for our ability to speak and consume food and serves as a gateway to the rest of the body. Poor oral health can lead to various systemic diseases, including cardiovascular problems, diabetes, and respiratory ailments. Consequently, the significance of dental care extends beyond a radiant smile; it is an integral component of comprehensive healthcare [1]. In recent years, a technological revolution has dramatically transformed the healthcare landscape. Innovations in medical imaging, diagnostics, and treatment methods have greatly enhanced patient care. Technology integration into healthcare has ushered in a new era, allowing for more accurate and efficient procedures and improved patient outcomes [2].

Artificial intelligence (AI) has emerged as a game-changing technology in healthcare, offering innovative solutions to long-standing challenges. Like other branches of healthcare, dentistry is undergoing a significant transition toward AI adoption. This transformation is driven by the potential of AI to revolutionize dental diagnosis, treatment planning, and patient engagement. The utilization of AI in dentistry promises to provide more precise, efficient, and personalized oral care [3]. This paper aims to explore the exciting frontier of AI in dental care and its implications for both professionals and patients. The following sections will delve into the various aspects of this new era of dental care, including its applications in diagnosis, treatment planning, and patient engagement. Additionally, the paper will address the ethical and privacy considerations accompanying AI integration in dentistry, as well as the challenges and future directions for this evolving field. Through a comprehensive examination of these topics, we seek to illuminate the potential of AI in ushering in a brighter future for dental care.

## Review

Artificial intelligence in dentistry

Artificial intelligence (AI) refers to developing computer systems capable of performing tasks that typically require human intelligence. These tasks encompass various activities, including problem-solving, learning, perception, reasoning, and decision-making. AI systems leverage advanced algorithms, machine learning, and data analysis to simulate human-like cognitive functions, enabling them to process and analyze large volumes of data at a rapid pace [4].

AI Applications in Healthcare and Dentistry

**Figure 1:**
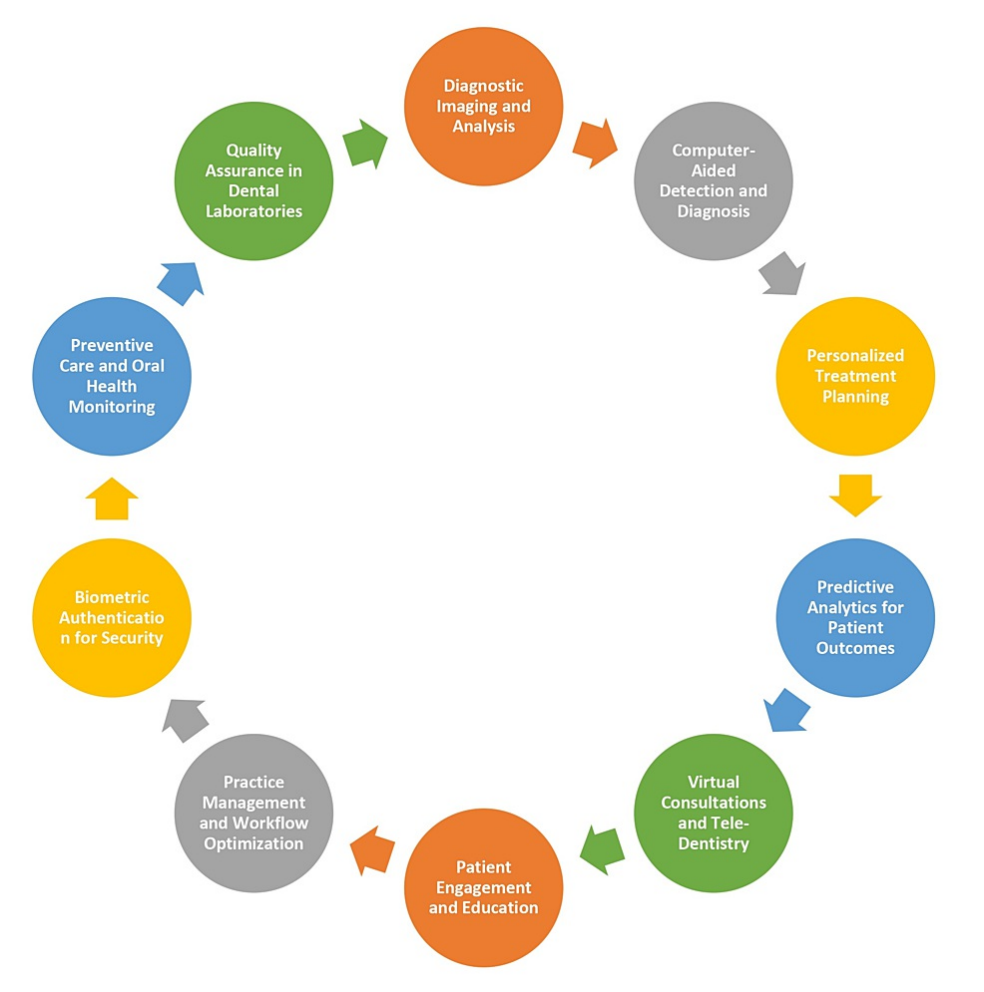
Application of AI in dentistry Image created by the authors AI: artificial intelligence

AI has found extensive applications across the healthcare sector, revolutionizing how medical professionals diagnose, treat, and manage patient care. In dentistry, AI is making significant strides in image analysis, diagnosis, treatment planning, and patient engagement. AI-driven systems can analyze dental records, radiographs, and intraoral images to detect abnormalities, assist in treatment decisions, and provide personalized recommendations. Moreover, AI applications extend to dental practice management, offering scheduling, billing, and patient communication tools to enhance patient experience. The application of AI in dentistry is described in Figure 1 [5].

Benefits of AI in Dental Care

Enhanced accuracy: AI in dentistry can process vast amounts of dental data, including X-rays, images, and patient records, with exceptional precision. AI algorithms can detect subtle abnormalities and patterns that may go unnoticed by human clinicians. This enhanced accuracy can lead to more precise and early diagnoses of dental conditions, ultimately improving patient outcomes. For example, AI can help identify cavities, gum diseases, or oral cancer at earlier stages when treatment is more effective [6].

Efficiency: AI automates various routine tasks in dental practices, such as data entry, image analysis, and appointment scheduling. This automation significantly reduces the time required for administrative work, allowing dental professionals to focus more on patient care. It also minimizes the chances of human error in tasks such as record-keeping, which can substantially impact the efficiency and accuracy of the practice [6].

Personalized care: AI systems can analyze a patient's dental history and health data to create highly personalized treatment plans. AI can recommend specific oral hygiene routines and preventive measures by considering individual patient needs. This tailored approach ensures that patients receive care well-suited to their unique circumstances, which can lead to improved oral health outcomes [3].

Early detection and prevention: AI-powered algorithms can detect oral health issues early. For instance, AI can identify signs of dental decay or periodontal disease before they become symptomatic. This early detection allows for timely intervention and the implementation of preventive measures. Preventing dental problems from progressing to more serious stages benefits the patient's health and can also reduce the long-term cost of treatment [7].

Improved patient experience: AI-driven patient engagement tools are vital in enhancing the patient experience. These tools can facilitate communication between patients and dental professionals, providing educational resources to help patients understand their treatment options and oral health. AI-driven chatbots or virtual assistants can also assist patients in scheduling appointments, sending reminders, and answering common queries, making it easier for patients to engage with their dental care. Ultimately, this improves patient satisfaction and compliance with recommended treatments and follow-up appointments [8].

Overview of AI Techniques Used in Dentistry

Machine learning: Machine learning involves training algorithms on extensive datasets of dental images and patient records. These algorithms learn to recognize patterns, anomalies, and correlations within the data. In dentistry, machine learning is frequently used for image analysis, such as identifying dental caries, bone fractures, or abnormalities in X-rays and other dental images. It can also assist in diagnosing conditions based on radiographic and clinical data, contributing to more accurate and efficient diagnoses [9].

Natural language processing (NLP): NLP focuses on the interaction between computers and human language. In dentistry, NLP can understand and interpret textual information in dental records, patient histories, clinical notes, and reports. It enables AI systems to extract meaningful insights from unstructured text, which can aid in understanding a patient's medical history, treatment plans, and progress. NLP can also help automate documentation and extract valuable information from large volumes of text-based data [10].

Computer vision: Computer vision is a field of AI that enables machines to process and interpret visual information. In dentistry, computer vision techniques are crucial for analyzing radiographs, intraoral images, and other visual data. AI systems can use computer vision to identify and assess dental conditions such as cavities, gum diseases, or abnormalities in the oral cavity. Additionally, it can track the progress of orthodontic treatments and monitor changes in oral health over time [11].

Predictive analytics: AI-driven predictive models in dentistry are essential for forecasting various aspects of patient care and dental practice operations. For instance, predictive analytics can estimate patient outcomes, predict disease progression, and assess the likely success of different treatment options. This helps tailor treatment plans to individual patients, make decisions based on data-driven insights, and improve the overall quality of care. In addition, predictive analytics can assist in optimizing appointment scheduling, resource allocation, and inventory management within a dental practice, thereby enhancing operational efficiency [12].

AI in dental diagnosis

AI-Driven Dental Imaging and Radiography

Image enhancement: AI algorithms can enhance dental images by improving the clarity and visibility of details. This can be particularly valuable in dental radiography, where subtle anomalies might not be easily discernible in standard images. Dental professionals can use AI for image enhancement to identify potential dental issues more effectively, even in challenging cases. Enhanced images can reveal finer details, aiding in the early detection and diagnosis of dental conditions [13].

Image analysis: AI's image analysis capabilities are crucial in automating the identification of common dental conditions. For example, AI can be trained to recognize and diagnose cavities, periodontal diseases, impacted teeth, and other dental anomalies in radiographs and intraoral images. This improves the speed and accuracy of diagnoses and ensures that dental professionals do not overlook any important findings, contributing to better patient care and outcomes [7].

Automation: AI can assist in automating various aspects of the radiographic process. This includes capturing images, positioning X-ray equipment, and ensuring image quality. Automation reduces the workload on dental staff and minimizes the potential for human error. By maintaining consistent imaging techniques and quality across different patients, AI can help produce more reliable diagnostic images and improve patient care [14].

Computer-Aided Detection and Diagnosis of Dental Conditions

Cavity detection: AI-powered systems excel at accurately detecting and quantifying dental cavities. They can identify cavities at their earliest stages, often before they are visible to the naked eye or even on conventional X-rays. Early detection is crucial because it enables timely intervention and treatment, preventing the progression of cavities to more extensive and severe dental problems. By using AI for cavity detection, dentists can provide more proactive and effective patient care, ultimately preserving oral health [15].

Periodontal disease assessment: AI algorithms can assess the severity of periodontal diseases by analyzing dental images and clinical data. This analysis helps dentists determine the appropriate treatment plans and monitor the condition's progression over time. The early assessment and monitoring of periodontal diseases are essential for preventing further damage to the gums and supporting structures, potentially reducing the need for more invasive treatments. AI provides a more comprehensive and accurate periodontal health evaluation [16].

Oral cancer screening: AI tools play a significant role in the early detection of oral cancer. By analyzing images of oral lesions, including tissue discolorations or irregularities, AI can help identify potential signs of oral cancer. Early detection is critical for improving the chances of successful treatment and potentially saving lives. AI-based oral cancer screening tools can be valuable to routine dental check-ups, providing an extra layer of vigilance in monitoring patients' oral health [17].

Early Detection and Prevention of Dental Diseases

Analyzing patient records: AI can efficiently analyze extensive patient histories and records to identify risk factors associated with oral health. By mining this data, AI can recognize patterns and correlations that may not be apparent through manual examination. This information allows dental professionals to offer personalized preventive recommendations to individual patients. For instance, if AI identifies a patient's susceptibility to gum disease due to their medical history and habits, it can suggest tailored preventive measures, such as more frequent cleanings or specific oral hygiene practices [18].

Predictive analytics: AI-driven predictive models are instrumental in forecasting disease progression within preventive dentistry. These models analyze a patient's historical data and predict how their oral health may evolve over time. This assists dental professionals in designing treatment plans that address potential issues before they become more complicated. By utilizing predictive analytics, dentists can intervene early and implement preventive measures, thus reducing the risk of severe dental problems in the future [7].

Patient education: AI can significantly influence patient education regarding oral health and hygiene. AI-driven systems can provide patients with personalized information, advice, and recommendations based on their dental records and specific needs. This encourages patients to adopt preventive measures and fosters better compliance with dental recommendations. For example, AI-powered educational tools can send automated reminders for regular check-ups, explain the importance of specific oral care routines, and answer patient queries, all of which contribute to a more informed and engaged patient population [19].

AI in treatment planning

AI-Assisted Treatment Planning and Decision Support

Data analysis: AI excels at sifting through large volumes of patient records, dental images, and other relevant data to extract meaningful insights. By analyzing this data, AI can identify the most suitable treatment options for individual patients based on their conditions and medical histories. For example, when presented with a patient's dental records and X-rays, AI can help identify the most appropriate treatment approach, considering the severity of dental issues, patient preferences, and prior treatment success rates. This data-driven decision-making improves the accuracy of treatment planning and ensures that patients receive care tailored to their unique needs [20].

Evidence-based guidance: AI algorithms leverage extensive datasets and clinical research to recommend treatment strategies that align with best practices and evidence-based clinical guidelines. AI systems can stay up-to-date with the latest developments in dentistry, ensuring that the guidance provided aligns with the most current knowledge. By drawing on this wealth of data and research, AI can assist dental professionals in making informed and evidence-based decisions. This enhances the quality of care and reduces the chances of outdated or suboptimal treatment approaches [3].

Real-time support: Dental professionals can access AI-driven decision support systems during patient consultations, facilitating real-time treatment planning discussions. AI can provide valuable insights, suggest treatment options, and offer information to the dentist and the patient. This real-time support enhances the quality of care by ensuring that dental professionals have the most up-to-date information during patient interactions. It also helps make informed decisions, answer patient queries on the spot, and foster a more collaborative approach to treatment planning [21].

Customized Treatment Plans for Patients

Tailored interventions: AI excels in generating treatment plans highly tailored to individual patients. By analyzing a patient's unique needs, preferences, and health history, AI can propose treatments that are not only effective but also patient-centered. For example, suppose a patient has specific medical conditions or sensitivities, in that case, AI can suggest treatment options that consider these factors, ensuring that the chosen interventions are safe and suitable for that particular patient. This level of personalization enhances the quality of care and increases patient satisfaction [22].

Treatment alternatives: AI can present patients with various treatment options, complete with information on the associated benefits and risks. Patients can then make more informed decisions about their oral health and treatment plans. AI can explain the pros and cons of each option, as well as the expected outcomes and potential side effects. This informed decision-making empowers patients to choose the best treatment with their preferences, budget, and long-term oral health goals, fostering a sense of agency and participation in their care [12].

Multidisciplinary collaboration: AI can facilitate multidisciplinary collaboration in complex cases requiring input from various dental specialists. Different dental professionals, such as orthodontists, oral surgeons, and periodontists, can work together on comprehensive treatment plans. AI can streamline communication and data sharing among these specialists, ensuring that each aspect of the treatment plan is well-coordinated. This collaboration can result in more efficient and effective care for patients with complex oral health issues, as it leverages the expertise of multiple dental disciplines to provide a holistic and integrated approach to treatment [23].

Predictive Analytics in Dental Treatment

Outcome prediction: AI can estimate the likely outcomes of dental procedures, providing valuable insights to patients and practitioners. For patients, this means understanding what to expect from a particular treatment, including potential risks and benefits. This informed decision-making empowers patients to make choices that align with their preferences and goals. For dental practitioners, outcome prediction helps in treatment planning by considering various factors, such as the patient's oral health and medical history and the chosen procedure. By relying on AI-generated predictions, dental professionals can design more effective and patient-centered treatment plans, improving patient satisfaction and successful treatment outcomes [24].

Disease progression modelling: AI algorithms are adept at creating models to predict how dental conditions may evolve over time. These models consider disease history, patient demographics, and environmental influences. By forecasting disease progression, dental professionals can intervene early to prevent or mitigate worsening conditions. For instance, in the case of periodontal disease, AI can predict how the disease may advance and recommend personalized monitoring and preventive measures. This proactive approach enhances patient care by addressing potential issues before they become more complicated, ultimately preserving oral health [25].

Treatment success assessment: AI can assess the success of ongoing dental treatments by comparing the progress against predefined criteria and benchmarks. This assessment helps in identifying whether a treatment is achieving its intended goals. If AI detects deviations or potential issues, it can suggest adjustments to the treatment plan or alternative approaches. This real-time feedback and support are invaluable in maintaining treatment efficacy and ensuring patients receive the best care. By continuously monitoring treatment success, dental professionals can make data-driven decisions, improving the likelihood of successful outcomes and reducing the risk of treatment failure or complications [6].

Patient experience and engagement

AI-Driven Patient Education and Communication

Educational content: AI can generate informative and easily understandable educational materials, such as videos, articles, and interactive presentations, to educate patients about dental conditions, treatments, and preventive care. This content can be tailored to each patient's specific needs and preferences, ensuring that they have access to accurate and personalized information. By delivering educational content, AI empowers patients to make informed decisions about their oral health and encourages them to maintain good dental hygiene proactively. Informed patients are more likely to comply with treatment recommendations and adopt preventive measures, leading to better oral health outcomes [26].

Interactive chatbots: AI-driven chatbots are invaluable tools for engaging with patients in real time. They can answer questions, provide information, and guide patients through various aspects of oral health, including posttreatment care. These chatbots offer a convenient and immediate source of patient support, enhancing patient engagement and satisfaction. Chatbots can be available around the clock to provide information, alleviate concerns, and offer assistance, thereby improving the overall patient experience and reducing the burden on dental staff for routine queries [27].

Appointment reminders: AI-powered systems can send automated reminders and notifications to patients, helping reduce no-shows and improving appointment adherence. These reminders can be customized to the patient's preferred communication method, such as SMS, email, or phone calls. By leveraging AI for appointment reminders, dental practices can better manage their schedules and optimize the utilization of resources. Patients benefit from timely notifications, ensuring they do not miss important appointments essential for preventive and ongoing dental care [28].

Personalized Oral Health Recommendations

Tailoring advice: AI systems excel at analyzing a patient's dental records, habits, and risk factors to create specific recommendations for maintaining oral health. These recommendations can include dietary suggestions, hygiene routines, and lifestyle modifications. By considering individual patient data, AI can generate personalized advice that is more relevant and more likely to be followed. For instance, if a patient has a history of enamel erosion, AI can recommend dietary changes to reduce acid exposure. This personalized approach ensures that patients receive guidance that aligns with their unique circumstances, increasing the likelihood of better oral health [29].

Preventive measures: AI can identify potential oral health risks by analyzing patient data and suggesting targeted preventive measures. This proactive approach ensures that patients maintain their oral well-being, such as recommending more frequent dental check-ups for individuals with higher risk factors or suggesting using specific oral care products. By leveraging AI to predict and prevent oral health issues, dental professionals can intervene early, potentially reducing dental problems and minimizing the associated patient costs and discomfort [7].

Customized treatment plans: When dental treatment is required, AI can propose treatment plans tailored to patients' unique needs and preferences. For example, if a patient needs a dental crown, AI can recommend materials and procedures that align with the patient's aesthetic preferences and budget. This personalization enhances patient satisfaction and ensures that treatment plans are effective and harmonized with the patient's goals and expectations. Customized treatment plans contribute to a more patient-centered approach to dental care [30].

Improved Patient Compliance and Follow-Up Care

Reminders and alerts: AI can send automated reminders for various aspects of oral care, such as follow-up dental appointments, medication schedules, and oral hygiene routines. These reminders ensure that patients remain consistent with their care plans and do not miss important appointments or treatments. By keeping patients informed and on track, AI helps improve adherence to recommended care regimens, resulting in better oral health outcomes. Automated reminders reduce the risk of missed appointments and support patients in maintaining their oral hygiene and treatment schedules [31].

Progress tracking: AI can assist patients in monitoring their oral health progress over time. This feature lets patients see the benefits of adhering to recommended care routines and treatments. By tracking improvements in oral health, patients can gain motivation and confidence in their chosen care plans. This visual feedback can reinforce positive behaviors and encourage patients to follow their dental professional's recommendations, contributing to better long-term oral health [7].

Telehealth options: AI can facilitate virtual consultations and follow-up care, making it more convenient for patients to seek advice and treatment guidance. This is particularly valuable for minor concerns or postoperative check-ups where in-person visits are unnecessary. Telehealth options save patients time and may reduce the need for physical visits to the dental clinic. Additionally, it can be an essential tool in emergencies, allowing patients to receive timely advice and support from their dental professionals without needing immediate in-person care. Telehealth options enhance patient convenience and access to dental services, especially when physical visits are challenging [32].

Ethical and privacy considerations

Data Security and Patient Privacy in AI-Powered Dentistry

Data protection: AI in dentistry relies heavily on collecting and analyzing sensitive patient data, including medical records and diagnostic images. Robust data protection measures are essential to ensure patient privacy and maintain data integrity. These measures include safeguarding against unauthorized access, data breaches, and data leaks. Data protection is a matter of regulatory compliance and an ethical responsibility to protect patients' sensitive information. Dental practices and AI developers must implement strict access controls, secure data storage, and conduct regular security audits to identify and address potential vulnerabilities [6].

Anonymization and encryption: Anonymization and encryption are key techniques to protect patient privacy in AI applications. Anonymization involves removing or altering personally identifiable information (PII) from patient data to ensure that the data cannot be linked to specific individuals. Strong encryption protocols secure data when it is transmitted or stored. These techniques make it extremely difficult for unauthorized parties to access or decipher sensitive patient information, reducing the risk of data breaches. Implementing anonymization and encryption is crucial when sharing data with AI systems or transferring data between healthcare providers and AI developers [33].

Consent and transparency: Patients have a right to know how their data will be used, especially regarding AI-powered healthcare applications. Dental practitioners and AI developers must be transparent about collecting, storing, and utilizing patient data for AI purposes. Patients should provide informed consent, understanding and agreeing to how their data will be used in AI applications. This transparency and consent comply with legal and ethical standards and build trust between patients, dental professionals, and AI developers. It ensures that patients know AI's potential benefits and uses in their healthcare and can make informed decisions about their participation [34].

Ethical Concerns and AI in Healthcare

Bias and fairness: AI algorithms can inadvertently inherit biases in their training data, leading to discriminatory outcomes in healthcare. Dentists and AI developers must be vigilant in identifying and mitigating bias to ensure equitable care for all patients. This involves continuously monitoring AI systems for biases and taking corrective actions when bias is detected. Having diverse and representative training data and employing fairness-aware algorithms that minimize bias in AI-driven decisions are essential. Ensuring fairness in AI applications in dentistry is a legal and ethical imperative and essential for providing equitable healthcare to all patients [35].

Accountability and transparency: The responsibility for AI-driven dental care must be clearly defined. Dental professionals and AI developers should establish mechanisms for transparency and accountability in AI systems. This includes tracking and auditing AI decisions and outcomes, identifying the responsible party in case of errors or adverse events, and having mechanisms to address issues promptly. Transparency is crucial to building trust in AI systems and ensuring that patients and healthcare providers understand how decisions are made and who is accountable when things go wrong [36].

Informed decision-making: Patients should be aware of AI systems involved in their care. They should have the right to opt out or seek human consultation. Informed decision-making means that patients understand how AI is used in their treatment and can choose whether to include AI recommendations in their care plan. This empowers patients and respects their preferences, ensuring they participate actively in healthcare decisions. Informed consent and the option for human consultation are important elements of patient-centered care in the context of AI [37].

Regulatory and Legal Aspects

Compliance with regulations: AI-powered dentistry must adhere to established regulations and legal frameworks, such as the Health Insurance Portability and Accountability Act (HIPAA) in the United States and other relevant laws in different countries. Compliance with regulations is critical to ensure that patient data is handled with the utmost care and by legal requirements. These regulations provide a framework for safeguarding patient privacy, securing sensitive health information, and defining how AI applications can be used in healthcare. Compliance is a legal requirement and crucial to maintaining patient trust and data security [38].

Certification and accreditation: Dental AI systems should undergo rigorous testing and receive appropriate certifications to ensure their safety, efficacy, and reliability. Regulatory bodies and professional organizations should establish guidelines for approving and monitoring AI applications in healthcare. Certification and accreditation processes help validate the quality and safety of AI systems, ensuring that they meet established standards and are suitable for use in clinical settings. This is essential to protect patient safety and to give dental practitioners confidence in the technology they employ [39].

Liability and malpractice: Legal frameworks must address liability questions when AI is involved in dental diagnosis or treatment planning. It is important to determine who is responsible in cases of errors or adverse outcomes. This includes defining the roles and responsibilities of dental professionals, AI developers, and healthcare institutions in the event of AI-related malpractice or adverse events. Addressing liability concerns ensures that patients have recourse in the case of negligence or harm while also providing clear guidelines for dental practitioners and AI developers regarding their legal obligations [40].

Challenges and future directions

Current Challenges in AI Implementation in Dental Care

Data quality and quantity: High-quality and extensive datasets are crucial for training AI models effectively. In dentistry, acquiring such data can be challenging due to privacy concerns and the limited availability of large-scale datasets. Patients' dental records are sensitive; sharing them for research can be subject to stringent privacy regulations. Dental professionals and AI developers must work to anonymize and aggregate data while maintaining data quality to ensure that AI systems are accurate and reliable [41].

Regulatory barriers: The regulatory environment for AI in healthcare, including dentistry, is complex and continually evolving. Navigating and ensuring compliance with these regulations can be a significant challenge. Regulatory bodies such as the FDA in the United States have specific requirements for approving and using medical AI applications. Staying up-to-date with evolving regulations and complying with them are essential but can be time-consuming and require significant legal and regulatory expertise [42].

Integration into practice: Integrating AI into dental practices requires investments in technology and staff training. While AI has the potential to enhance patient care and streamline operations, it often involves costs associated with purchasing and implementing AI systems, as well as training dental professionals to use them effectively. Smaller practices may find these investments challenging, and some dental professionals may resist incorporating AI into their established workflows. Successful integration may require change management strategies to ensure a smooth transition [6].

Bias and fairness: Mitigating bias in AI algorithms used for dental diagnosis and treatment planning is an ongoing challenge to ensure equitable dental care. AI systems can inherit biases from the data they are trained on, which can lead to unfair or discriminatory outcomes. Dental practitioners and AI developers must continually work to identify and address bias, employ fairness-aware algorithms, and ensure that AI applications provide equitable patient care. This involves continuously monitoring and refining AI systems to minimize bias and enhance fairness [24].

Research and Development in AI Dentistry

Advancements in image analysis: Ongoing research is dedicated to improving AI algorithms for the analysis of dental images. This includes radiographs, intraoral photographs, and 3D scans. The goal is to enhance the accuracy and efficiency of image analysis to detect various dental conditions, such as caries, periodontal diseases, and abnormalities. Advanced image analysis AI systems are designed to identify these conditions at their earliest stages, often before they are visible to the human eye or even conventional X-rays. This early detection can significantly impact treatment outcomes by allowing timely intervention, reducing the need for more invasive procedures, and improving patient outcomes [6].

Personalized treatment planning: Researchers are developing AI systems to create even more individualized and patient-centric treatment plans. These AI systems consider various factors, including genetic predisposition, lifestyle choices, and environmental influences. Considering these diverse variables, AI can provide highly personalized recommendations for preventive measures and treatment options. For instance, AI may analyze a patient's genetic data to predict their susceptibility to certain oral health conditions and suggest personalized strategies to mitigate those risks. This personalized approach to treatment planning aims to optimize patient outcomes and experiences by tailoring care to individual needs and preferences [5].

Tele-dentistry and remote monitoring: The development of AI-enabled remote monitoring tools is poised to revolutionize the accessibility of dental care, particularly in underserved areas or during public health crises. Tele-dentistry applications can connect patients with dental professionals remotely, allowing for consultations, follow-up care, and the monitoring of oral health conditions through video calls, images, and other data-sharing methods. AI plays a crucial role in these applications by helping remotely assess and monitor patients' oral health. This expands access to dental care and ensures that patients receive timely guidance and interventions, particularly when in-person visits are challenging or limited, as was the case during the COVID-19 pandemic [43].

Future Prospects and Advancements

AI-driven robotic dentistry: Integrating AI with robotic systems is poised to revolutionize dental procedures. AI-driven robotic dentistry can enhance precision and automation in tooth drilling, implant placement, and orthodontic adjustments. These robots can operate with precision that exceeds human capabilities, reducing the risk of human error and improving treatment outcomes. This technology can make dental procedures more efficient, less invasive, and less painful for patients, contributing to better patient experiences and outcomes [44].

Augmented reality (AR) and virtual reality (VR) in education: AI-powered augmented reality (AR) and virtual reality (VR) applications can potentially transform dental education and training. Dental students and practitioners can benefit from immersive experiences that simulate real clinical scenarios, providing a safe environment for learning and practicing various dental procedures. AI can enhance these experiences by providing real-time guidance and feedback to learners. This approach accelerates the learning curve and offers a dynamic and engaging way to train dental professionals, ultimately leading to higher competence and confidence [45].

Predictive analytics for public health: AI can be used for predictive analytics in public health. By analyzing vast datasets, AI can forecast oral health trends and identify at-risk populations. This information can be valuable for public health agencies and policymakers in developing more effective preventive programs and interventions. For example, AI can help design community-level preventive programs targeting specific demographics or geographic regions with a higher prevalence of oral health issues. These interventions can improve public health and reduce healthcare costs [46].

Global access to dental care: AI-driven tele-dentistry and portable diagnostic devices can expand access to dental care, even in remote or underserved regions. Tele-dentistry allows patients to consult with dental professionals remotely, reducing the need for physical visits. Portable diagnostic devices equipped with AI can aid in early disease detection and monitoring, particularly in areas with limited access to dental facilities. This technology democratizes dental care, making it more accessible to underserved populations and addressing oral health disparities on a global scale [47].

## Conclusions

In conclusion, integrating artificial intelligence (AI) into dental care marks the dawn of a transformative era. The profound impact of AI on dental diagnosis and treatment is indisputable, presenting myriad benefits. These range from elevating the precision of dental imaging and facilitating computer-aided detection and diagnosis to streamlining personalized treatment planning and predicting patient outcomes. These advancements can revolutionize dentistry, rendering it more efficient, patient-centric, and effective. However, it is imperative to emphasize the necessity for a collaborative synergy between AI and dental professionals. Specifically, it would be valuable to delve into concrete ways in which dental practitioners and AI can collaboratively ensure AI's responsible and ethical use in patient care. AI should be positioned as a complementary tool that enhances the expertise of dental practitioners rather than a replacement. By fostering a partnership between AI and dental experts, we can guarantee this technology's responsible and ethical application, upholding the highest standards of patient privacy and data security. This collaborative approach enhances patient care and contributes to a brighter and healthier future for dental practice, benefiting patients, practitioners, and the healthcare system at large.
